# Shared decision making when patients consider surgery for lumbar herniated disc: development and test of a patient decision aid

**DOI:** 10.1186/s12911-019-0906-9

**Published:** 2019-10-04

**Authors:** Stina Brogård Andersen, Mikkel Ø. Andersen, Leah Y. Carreon, Angela Coulter, Karina Dahl Steffensen

**Affiliations:** 1 Spine Surgery and Research, Spine Center of Southern Denmark, Lillebaelt Hospital - University Hospital of Southern Denmark, Øster Hougvej 55, Middelfart, Denmark; 2 Center for Shared Decision Making, Lillebaelt Hospital - University Hospital of Southern Denmark, Vejle, Denmark; 3 Faculty of Health Sciences, Institute of Regional Health Research, University of Southern Denmark, Odense, Denmark; 4 Norton Leatherman Spine Center, Louisville, KY USA; 5 Nuffield Department of Population Health, University of Oxford, Oxford, UK; 6 Department of Clinical Oncology, Lillebaelt Hospital - University Hospital of Southern Denmark, Vejle, Denmark

**Keywords:** Shared decision making, Patient decision aid, Spine surgery, Lumbar disc herniation

## Abstract

**Background:**

Shared decision making (SDM) is a systematic approach aimed at improving patient involvement in preference-sensitive health care decisions. Choosing between surgical or non-surgical treatment for lumbar disc herniation, can be difficult as the evidence of a superior treatment is unclear, which makes it a preference-sensitive decision. The objectives of this study was therefore to assess the degree of SDM and afterwards to develop and test a patient decision aid (PtDA) to support SDM during the clinical encounter between surgeon and patient, when patients choose between surgical and non-surgical treatment for Lumbar disc herniation (LDH).

**Methods:**

The study was conducted in four steps.

1. Assessment of the extent to which SDM was practiced in the spine clinic.
2. Development of a PtDA to support SDM.
3. Testing its usability and acceptability amongst potential users (patients).
4. Pilot-test of its usability in the clinical setting.

**Results:**

Results from our small baseline study (*n* = 40) showed that between a third and two-thirds of the patients reported not being fully engaged in a shared decision. A pre-designed template (*BESLUTNINGSHJÆLPER*™) was adapted to support the decision about whether or not to have surgery for LDH. Testing the prototype with patients led to minor refinements. A subsequent pilot test of its usability in a clinical setting achieved positive responses from both patients and clinicians.

**Conclusion:**

Our baseline study demonstrated that SDM was not universally practiced in the clinic. The PtDA we have developed was rated as acceptable and usable by both patients and clinicians for helping those with LDH choose between surgical or non- surgical treatment. This tool now requires further testing to assess its effectiveness.

**Electronic supplementary material:**

The online version of this article (10.1186/s12911-019-0906-9) contains supplementary material, which is available to authorized users.

## Background

Shared decision making (SDM) is a systematic approach aimed at improving patient involvement in preference-sensitive health care decisions. It is a process, wherein clinicians actively involve patients during the task of making decisions, based on best clinical evidence and patients’ informed preferences [1].

Most people with lumbar disc herniation (LDH) improve with nonsurgical treatment [2]. However, the Danish national clinical guidelines for nonsurgical treatment of recent onset lumbar nerve root compression recommend surgical evaluation if patients have ongoing severe pain and disability after 12 weeks of conservative care [3]. Apart from the presence of a neurologic deficit, there are no clearly defined indications for surgery. A variety of factors, such as level of pain, walking capacity, working ability or other functional limitations, can influence the judgment of the physician to advocate for surgery or not. Indications for surgery are thus assessed, for each particular patient, by the individual surgeon. Even though surgical patients show a more rapid recovery and relief of pain, the evidence of surgery being superior to conservative treatment in the long term remains inconclusive [4]. This underlines the concept that the choice of having surgical or non-surgical treatment for LDH is preference sensitive, meaning that treatment options should be offered and should matter to patients. A qualitative study by Andersen showed that the current lack of evidence for a superior treatment and the presence of uncertainties in risks and benefits for each treatment approach are important to discuss with patients with LDH [5]. Currently, there are no standard procedures used to actively involve patients in such decisions. SDM is required to accomplish the preference sensitive approach for patients with herniated disc.

Patient decision aids (PtDA) are tools designed to facilitate SDM, and are intended to complement counseling with health professionals [6]. Such a tool could help patients become involved in decision making by making the decision that needs to be made explicit, providing information about the advantages and disadvantages of treatment options (or tests), knowledge of treatment outcomes, and by clarifying personal values [6]. Decision aids compared to usual care seem to improve decision quality and patient knowledge regarding options and reduce their decisional conflict [6, 7].^.^ Some studies also show that PtDAs enable patients to be more actively involved in decision making, and improve the accuracy of risk perceptions when probabilities are included [6]. A few English tools have already been designed for patients with LDH [8–11]. However, these tools were designed to prepare the patients, prior to consultation with a physician and not, as intended in this project, to be used during the clinical encounter. No PtDAs have been designed for use in spine surgery in a Danish context.

Since SDM is not a new concept, it might already be adopted in spine surgery clinics. However several experts have found the task of implementing SDM in routine practice to be quite challenging [12–16]. Barriers include lack of a mutual understanding of SDM and the purpose and value of engaging patients in decisions [14]. An essential step before developing a PtDA is to evaluate the current extent of SDM. The objective of this study was to evaluate the degree of SDM in a spine surgery clinic, when patients were choosing between surgical and non-surgical treatment for LDH, and to develop and test a PtDA to support SDM in the clinical encounter between surgeon and patient.

## Methods

**Fig. 1:**
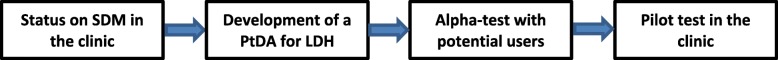
The four steps of the study

This paper describes four steps (Fig. 1). In the initial step, we did a baseline study, to evaluate to what degree SDM was implemented in the decision making process. The results of the baseline study showed a need to optimize SDM in the clinic. Patient decision aids can be useful to support SDM [17], therefore as a following step we decided to develop a PtDA to support SDM. In step three the usability and acceptability of the PtDA was tested systematically among potential users (patients) and finally in step four the usability was tested in the clinical setting. Each of the different steps involved different clinicians and patients, to ensure input and feedback from as many potential users as possible and thereby, development of a more applicable PtDA.

### Step 1 – baseline status on SDM practice

In order to investigate from a patient perspective whether main elements of SDM were already practiced, a survey was conducted in a spine surgery clinic. Danish versions of the questionnaires: CollaboRATE, Decision Quality Worksheet – for herniated disc v. 2 (DQW-HD) and Decisional Conflict Scale (DCS) were used [18–20] (Additional file 1). Forty patients with LDH and concordant MRI findings with possible indication for primary discectomy, assessed by an experienced spinal surgeon, returned the questionnaires. Thirty-nine out of forty completed both DQW-HD and DCS. Thirty-eight out of 40 completed CollaboRATE. All statistical analysis was carried out using STATA version 15 [21]. As all scales are long ordinal scales data were analyzed as non-parametric data and presented as median and range.

### Step 2 - development of a patient decision aid for LDH

**Fig. 2:**
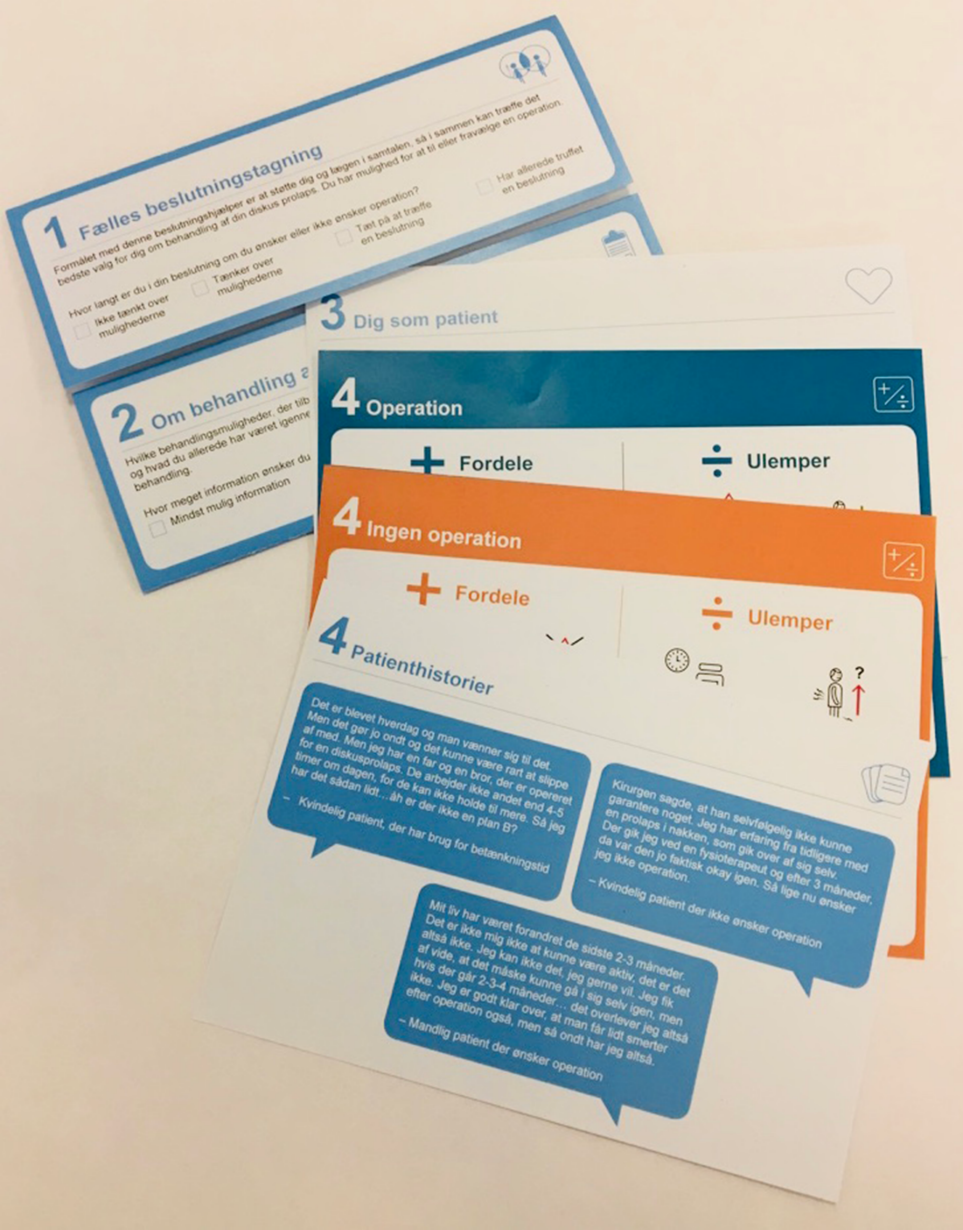
The PtDA [*BESLUTNINGSHJÆLPER™]* used in this study: a paper leaflet consisting of a frame with loose-leaf cards, called option cards, placed within

This project was developed in close cooperation with Centre for shared Decision Making at Lillebaelt Hospital. To support SDM in clinical settings the Centre has developed a generic PtDA, in Danish called *BESLUTNINGSHJÆLPER*™ in English a “Decision Helper” [22], according to the IPDAS criteria [23–25] and in collaboration with designers from Design School Kolding, Denmark. The *BESLUTNINGSHJÆLPER™* is a folder containing loose-leaf cards, called option cards (Fig. 2)”.The PtDA consists of five sections, as described by Olling et al. [22]: [1] the health care provider presents the PtDA and outlines the decision to be made [2]; the options are presented and the patient’s preference for level of information is assessed [3]; the patient’s preferences, fears and expectations are clarified and discussed [4]; different cards where information about advantages and disadvantages of the individual options, statistics, timelines and patient stories can be presented; and [5] assessment of readiness to make a decision, including the possibility of deferring the decision. In this current project the template was adapted to treatment choices for LDH and populated with specific content concerning LDH, the actual decision, advantages and disadvantages of each treatment, patient narratives etc.

The following methods were used to adapt the PtDA template to LDH:

- Two experienced spine surgeons and a medical spine specialist participated in ongoing interviews to develop a consensus about the options patients could choose from and the disadvantages and advantages of each option. As experts, they also helped to clarify the patient’s pathway at the spine center and were involved in discussions about how to present treatment outcomes.
- Semi-structured interviews with 14 patients were conducted to identify what could be important to patients with LDH when making the decision on whether or not to have surgery and to explore how the symptoms might affect them. The detailed method, material and results regarding the patient perspective is published previously [5].
- A systematic literature search was carried out in order to assess evidence-based risks and benefits of surgery versus non-surgical treatment (for further details please see Additional file 2).
- In order to present the risks of surgery for LDH in the PtDA, determination of the rate of severe complications after lumbar discectomy was evaluated. The risk profile was generated using local data from the Danish national surgical spine database (DaneSpine) on 2596 patients surgical treated for LDH. The data was collected from June 2010 to February 2017. Data on new onset neurological deficits and urinary disorders were reported by physiotherapists at follow-up consultations 1 month postoperative. Data on: thrombosis, embolism, urinary retention, perioperative infection, root injury or cauda equina reported by the surgeons at discharge, and data on: deep infection, thrombosis or embolism up till 3 months postoperative, reported by patients 1 year postoperative, were collected from the national spine surgery database DaneSpine.

### Step 3 - alpha-test with potential users

An alpha test of the adapted PtDA was conducted with potential users to determine acceptability and usability of the developed PtDA and whether the PtDA prepared patients for SDM. A method used by Stacey et al. was adopted where a structured interview guide was used followed by more open-ended clarifying questions [26]. The interview-guide was forward-back translated into Danish. Two items from the preparation for decision making scale were found irrelevant and removed from the interview guide. Ten participants, who were considered as candidates for surgery due to a LDH, were recruited from the Spine center. When information about the project was given and participation was accepted, a copy of both the PtDA and the interview guide were handed out and a date for the interviews was planned. Interviews took place either at the clinic or by phone. Every interview started by introducing the PtDA to the participant and talking them through it as if it was used in a consultation.

### Step 4 – pilot test in the clinic

To test the usability and feasibility in the clinic, two consecutive pilot tests were performed. First an experienced spine surgeon used the PtDA whenever a patient with symptoms and concordant MRI findings of LDH was referred for primary discectomy surgery. The consultations were observed by a researcher. After each consultation a brief evaluation of the consultation was made by the surgeon and the researcher. Any suggestions for changes in the PtDA were noted. During the 5 day period the PtDA was tested in six patient-surgeon consultations. Changes were made in the PtDA in agreement with identified issues in this first pilot test.

After the first pilot-test five other surgeons were introduced to the PtDA, and practiced their skills in a simulated consultation with a professional actress as a patient. Afterwards the surgeons were asked to test the PtDA in a real consultation. A short group discussion was made subsequently to identify if there were any final changes needed. The discussion was video recorded and led by the main researcher of the project. Remarks that the participants had expressed or issues that observations had revealed during the test period formed the basis of the discussion.

## Results

### Step 1 – baseline status on SDM practice

Forty-four patients were asked to participate in the survey about the current degree of SDM in the spine surgery clinic. Thirty-nine patients seen by 8 different surgeons returned the questionnaires. The result of the survey is shown in Table 1. Twenty-five out of 39 patients (64%), were according to the DQW - decision process score, not fully engaged in the decision-making process (had a score lower than 100). However, when a top score^1^ was calculated for CollaboRATE 63% seems to report a maximum score of SDM. A sub analysis was performed of the two items in the DQW questionnaire concerning whether both surgery and non-surgical treatment respectively were presented to the patient as possible treatment option. This analysis revealed that surgical treatment was suggested to all patients, but 36% of the patients reported that they were not informed about non-surgical options.

**Table 1:** Shared Decision Making measured by DQW-HD, DCS and CollaboRATE

	Result	Interpretation
DQW-HD (knowledge), (mean (SD))	42.69 (19,3)	0 = No knowledge 100 = Best possible knowledge
DQW-HD (Decision process), (mean (SD))	76.39 (22,75)	0 = No engagement in the decision 100 = Best possible engagement in the decision
DCS, (mean (SD))	18 (12,41)	0 = No Decisional conflict 100 = Extreme decisional conflict
DCS, (n (%)): score < 25 score 25–37.5 score > 37.5	25 (64.1) 11 (28.2) 3 (7.7)	Scores lower than 25 are associated with implementing decisions. Scores beyond 37.5 are associated with decision delay or feeling unsure about the decision.
CollaboRATE, (mean (SD))	8.45 (1,01)	1 = No effort was made 9 = Every effort was made
CollaboRATE top score (%)^a^	63,16	0 = No SDM 100 = Gold standard of SDM

The numbers presented in this table indicates the level of SDM before testing a PtDA in the current clinic. The scores of HQW-HD and DCS are calculated on *n* = 39 and CollaboRATE on *n* = 38

^a^The topscore is the number of patients who have responded 9 to all three of the CollaboRATE items, meaning every effort was made to make a shared decision

Our conclusion from this small baseline study was that some degree of SDM was already taking place in the spine surgery clinic for at least a third of the patients, but there was considerable room for improvement.

### Step 2 – development/adaption of patient decision aid for LDH

- To decide on elements to be incorporated in the PtDA clinicians pointed out that the majority of the patients seen by a surgeon had previously received non-operative treatment. The options presented in the PtDA therefore, were surgery or no surgery. No surgery would often be a “wait and see” approach but could be supplemented by pain-medication or in rare cases a rehabilitation program if this had not been tried previously.
  The clinicians were also asked to list the pros and cons of the two options. Those are presented in Table 2.
- The semi-structured patient-interviews revealed several issues that could be important to address in the consultation. A card presenting some of these was added to the PtDA for the purpose of facilitating a dialogue about what matters most to the patient. We found this very important, as it was not possible to send a preparation letter to the patients, so that they could consider their values ahead of time. The interviews showed that patients were limited in their everyday lives on different levels. Symptoms could disturb their sleep, limit their working capacity, and hinder social activities or continuation of sport activities. Even short walking distances could be hard to conquer because of the very intense nerve pain. The interviews led us to develop three different narratives for presentation in the PtDA outlining different patients’ stories about what they had chosen - to have or not to have surgery - and emphasizing the need for time to consider their choices.
- The literature review helped to assess the effects of surgery versus non-surgery treatment. It was difficult, to find any studies with the exact options presented in our PtDA. Only one high quality RCT by Peul et al. was identified comparing the efficacy of early surgery vs. prolonged conservative care [27]. They concluded that surgery in patients with sciatica provides better short term pain relief of leg pain and a faster recovery. This was also confirmed in a systematic review by Jacobs et al. [4]. Buttermann et al. showed that significantly fewer surgery patients used medication at 1–3 months follow-up compared to a group of patients treated with epidural steroid injections [28]. In the long term, no studies found any significant differences between groups on any outcomes [4]. As the evidence is sparse, presenting uncertainties in a PtDA is important for both this project and for all health care decisions per se. Therefore non-committal expressions like: “Possibly quicker pain relief” or “Perhaps your symptoms will decrease” were used to describe potential disadvantages and advantages.
- A risk calculation on data collected at the involved spine center showed that 129 of 2596 patients had experienced at least one severe complication after discectomy.

**Table 2:** Demographic characteristics of participants in the alpha-test (*n* = 10)

Age	
30–39	3
40–49	1
50–59	2
> 60	4
Gender	
Male	3
Female	7
Highest level of education	
College degree	3
Vocational education	6
Unknown	1
Experience with lumbar disc herniation	
Surgery for lumbar disc herniation	5
No surgery	4
Relative of patient with lumbar disc herniation	1

In total, eight consecutive versions of the PtDA-frame and 12 versions of the options cards were drafted. Version 1–4 of the frame and version 1–7 of the option cards were preliminary versions, which were changed due to designer’s observations from ongoing interviews, layout related changes, test of different statistical presentations of risk and expected outcome, reprioritization in relevant disadvantages and advantages etc. Frame version 4 and option cards version 7 were tested with potential users. Minor changes from the alpha-test and the following pilot tests led to the final versions.

### Step 3 - alpha-test with potential users

Ten out of twelve participants completed the interviews. Demographic characteristics are presented in Table 3. One was too tired to answer all the questions and one could not read Danish properly. All participants found that the amount of information was just about right and overall balanced, with no bias towards having or not having surgery. Two out of ten informants did not find the icons and font fully readable; the reasons are explained below. The space for data entry was found adequate (10/10), the words made sense (10/10) and the length of the PtDA was appropriate (10/10). Overall the informants thought that the PtDA would fit in the clinical consultation; three suggested some alterations and one did not answer that specific question. All, but one, were willing to use the PtDA. Further findings of acceptability, usability and the PtDA’s ability to prepare the patient for decision-making are presented in Table 3.

**Table 3:** Participants’ comments on the decision aid (alpha test)

Acceptability and usability	Stronglyagree	Agree	Neither agree or disagree	Dis-agree	Stronglydis-agree
The purpose of the decision aid is clear	4	6			
It is clear that I can chose how much information I want	4	5	1		
It is clear that I can state/discuss what worries me most	2	6	1	1	
The benefits and harms of each option is clear to me	7	3			
It is clear that I have to make a choice	5	5			
Preparation for decision–making	Not at all	A little	Some-what	Quite a bit	A great deal
Help patient recognize a decision needs to be made			1	4	5
Prepare patient to make a better decision	1			5	4
Help patients think about the pros/cons of options				4	6
Help patients think about which pros/cons are most important				8	2
Help patients know that the decision depends on what matters most to them		1		7	2
Help patients to organize their own thoughts about the decision			1	4	5
Help patients think about how much they want to be a part of the decision		1	1	2	5
Help patients ask questions and discuss them with the doctor.		2	1	4	2

### Qualitative findings

One participant was not convinced about the benefits of the PtDA and its use in the clinical encounter with the surgeon. However, the remaining patients liked the idea of the PtDA, and thought it would be helpful in the decision-making process. It was clear from the interviews that the PtDA could not stand alone, and a surgeon/health care professional was needed to facilitate its use in the consultation and elaborate some of the sections in the PtDA. The interviews provided constructive feedback for improvement of the PtDA. Suggestions led to changes if feasible. Two patients mentioned that the blue text and the small font were difficult to read. One patient suggested fewer option cards, as eight cards were hard to cope with. However, a suggestion about handing out the PtDA before a given consultation was not possible due to the way this clinic is organized. Further comments and suggestions are listed in Table 4.

**Table 4:** Participants’ suggestions for improving the decision aid (alpha-test)

*Lines under the text in section 3, where important personal issues could be noted.*	
*Use the inner side of the frame (section 4) and the back.*	
*Add worries about the future in card 3.*	
*A good help afterwards to support the decision made.*	
*Nice to have the information visualized.*	
*Miss the timeline of no-surgery treatment.*	
*A bit too long text in section 3 of the frame.*	
*How fast is “faster withdrawal from pain medication”?*	

### Step 4 – pilot test in the clinic

After the alpha-test two different pilot tests were performed in the clinic. Both of them showed a steep learning curve for clinicians and difficulty in breaking old routines. It also taught us basic things like how to present the tool to the patient both verbally and physically. The tests led to a reduction in the number of option cards, with two information cards describing the two options, being removed. A discussion about whether rehabilitation after surgery was a disadvantage of surgery led to removal of this statement from the card listing disadvantages of surgery. Lifelong training is recommended for both surgical and non-surgical patients; the argument was therefore, that many patients found it positive that they were offered rehabilitation regardless of their choice. During the test some surgeons raised concerns that: 1) they would forget important information that was part of their former routine and 2) it would affect the relationship and interaction between the surgeon and the patient. However, they considered that using a PtDA was a matter of good practice, as was being confident in its use.

### Readability index

The PtDA designed in this study is primarily a visual aid, made by designers, with very little text and short sentences (Fig. 2). The readability index of the overall text, can be interpreted as an easy text to read (LIX = 32). What also needs to be taken into account is that the PtDA is used in the consultation, so the content is also explained to the patient by the clinician.

## Discussion

The baseline study showed us the need for additional support for SDM in the surgical clinic. We are aware of the dissimilar findings concerning the extent of SDM when we compared the different ways to score or analyze the questionnaires. This becomes clear for instance when we look at the DCS score. The mean score is 18 and therefore below the threshold of 25, which is associated with implementation of SDM [20]. However, when we look at the score of each individual patient one third scored above 25. This indicates that the principles of SDM were not fully integrated in routine practice [20]. The CollaboRATE and the DQW-knowledge score supported this finding. The CollaboRATE topscore showed that one third of the patients did not reach gold standard of SDM and the mean DQW-knowledge score told us that on average patients only answered two out of five questions correctly. Information to the patients about the options could therefore be improved. Compared to Sepucha’s study [29] the mean DQW-process score found in our study seems to be fairly good. The method used in the baseline study may not capture all the aspects of SDM and does not meet a dyadic approach, as the physicians’ behavior or skills are not directly assessed. Having used an observational approach could have helped us to accomplish this.

### Presenting evidence and defining advantages and disadvantages of treatment

When patients make decisions about treatment, presenting the evidence is crucial, including presenting the absolute risk or expected outcome of a treatment. However, in surgery a common challenge exists because of an absence of high quality low biased RCT studies [4]. There is often a large extent of cross-over between surgery and non-surgery groups, and patients who already have a preference for a treatment-option might reject to participate [30]. Patients with LDH, who are offered surgery at the participating clinic often present with ongoing severe symptoms for 10–12 weeks or more. The majority are on pain medication, often opioids. In the literature search we were not able to find evidence based exact numbers to present how often symptoms would decrease spontaneously, the risk of permanent nerve-damage with no surgery or how long pain could be expected to last. We decided to include all these factors in the PtDA anyway, emphasizing the need for clinicians to make it clear to the patient that the likelihood of specific outcomes is sometimes unknown. The literature search was limited to RCT’s. This could potentially leave out cohort studies, which present relevant numbers of risk of treatment, but oppositely it ensured that the studies included patients with actual indication for surgery.

Another challenge was how to present the advantages and disadvantages of the options. What could be seen as an advantage to some patients or clinicians could be a disadvantage to others. For example, some saw rehabilitation as time-consuming and disruptive to everyday life or work, while others saw it as very positive.

Use of a pre-designed PtDA template has its pros and cons. The template required adherence to IPDAS criteria, ensuring compliance with a quality standard, but the design had certain limitations, including a fixed number of advantages and disadvantages, and a lack of flexibility due to relevant design considerations making it harder to adapt the layout.

### Testing content and usability of the decision aid

There is no standard method of how to test and validate a decision aid. In our study we used a questionnaire with closed questions and fixed response categories in an interview setting, which had some methodological challenges [26]. Patients sometimes found it difficult to reply to the closed response categories, and often preferred to explain instead of categorizing. However, it was also strength of the method that the patients could elaborate their answers through more open-ended questions. Some patients found a few of the questions difficult to understand or duplicative.

The usability of the PtDA was confirmed in this clinical setting, but we were unable to test its generalizability to other spine surgery clinics. However, the PtDA-template has, also been adapted and tested with two other patient groups, in two different departments, demonstrating good acceptability and usability amongst both patients and clinicians [22].

Even though patients and surgeons found the PtDA feasible, there was still a concern that it could interrupt daily routines. This has been highlighted before as a potential barrier to the uptake of PtDAs [31].

### Future steps

Using a pre-designed PtDA has some limitations as mentioned earlier. Apart from the fact, that it is not possible to change the lay-out, a paper-based PtDA might have a limited capacity on generalizability, transparency, data exchange, etc. This, seen in the light of increased use of electronic health information systems and health data, could speak in favor of developing an electronic version of the “BESLUTNINGSHJÆLPER™”. It is however, worth noting that the patients involved in development of the generic frame, actually preferred a tangible paper version of a PtDA, rather than an electronic-based app or web-system [22]. The idea of using a thoroughly tested generic template, developed according to the IPDAS criteria is favorable, as it has the potential to secure the quality of future PtDA’s.

## Conclusion

Knowing that the evidence favoring either surgical or non-surgical treatment for LDH is inconclusive, the choice of having surgical or non-surgical treatment for LHD is preference sensitive. SDM could be a way to achieve a preference sensitive approach. As a part of this project a baseline study was conducted, demonstrating that SDM was not fully integrated in the clinical encounter when patients with LDH choose between surgical or non- surgical treatment. In this study a generic PtDA template proved to be adaptable to these patients and was rated as acceptable and usable by both patients and clinicians.

What still needs to be confirmed is evidence of the tool’s ability to optimize the SDM-process. A randomized controlled trial is planned to test the effectiveness of the current LDH PtDA.

## Additional files

Additional file 1: Description and illustration of questionnaires used in this study (DOCX 670 kb)

Additional file 2: Description of Litterature search string and strategy (DOCX 21 kb)

## Data Availability

The datasets generated and/or analyzed during the current study are not publicly available due to individual privacy could be compromised, but are available from the corresponding author on reasonable request.

## Abbreviations

DQW-HD: Decision Quality Worksheet for Herniated Disc
IPDAS: International Patient Decision Aid Standards
LDH: Lumbar Disc herniation
MRI: Magnetic Resonance Imaging
PtDA: Patient Decision Aid
RCT: Radomized Controlled Trial
SDM: Shared Decision Making

## Footnotes

1 The topscore is the number of patients who have responded 9 to all three of the CollaboRATE items, meaning every effort was made to make a shared decision.
