# Supplementary material for: Shared decision making when patients consider surgery for lumbar herniated disc: development and test of a patient decision aid
Source: BMC Med Inform Decis Mak. 2019 Oct 4;19:190. doi: 10.1186/s12911-019-0906-9 (PMC6778367; doi:10.1186/s12911-019-0906-9)

**Appendix I Description and illustration of questionnaires used in this study**

**Decision Quality worksheet – for herniated disc** (DQW-HD v.2.0) is a disease-specific questionnaire measuring the extent to which patients are: 1) informed, 2) involved in the decision making process and 3) receive treatments that match their goals and preferences. In this study, for practical reasons, only the first two dimensions were used. Each dimension has a separate total score ranging from 0-100, [0 = no knowledge or no involvement in the decision; 100 = best possible knowledge or best possible involvement in the decision]. In the Danish version some of the answers in the section “Facts about herniated disc” are adapted to results found in Danish research-data, but all questions are the same.

Decision Quality Worksheet for Herniated Disc v. 2.0. ©Massachusetts General Hospital, 2010, updated 2012. https://mghhealthdecisions.files.wordpress.com/2018/06/hd_dqi_sv.pdf. Accessed December 6, 2018.


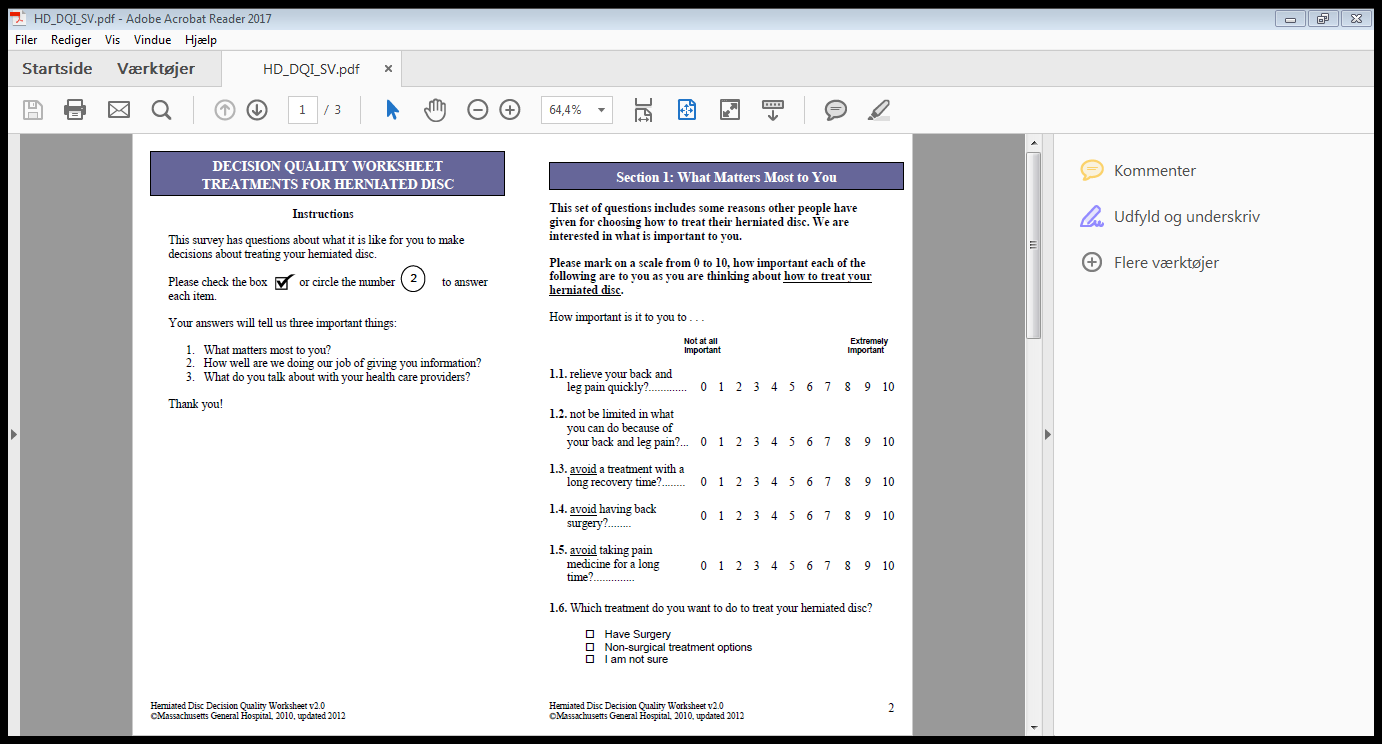


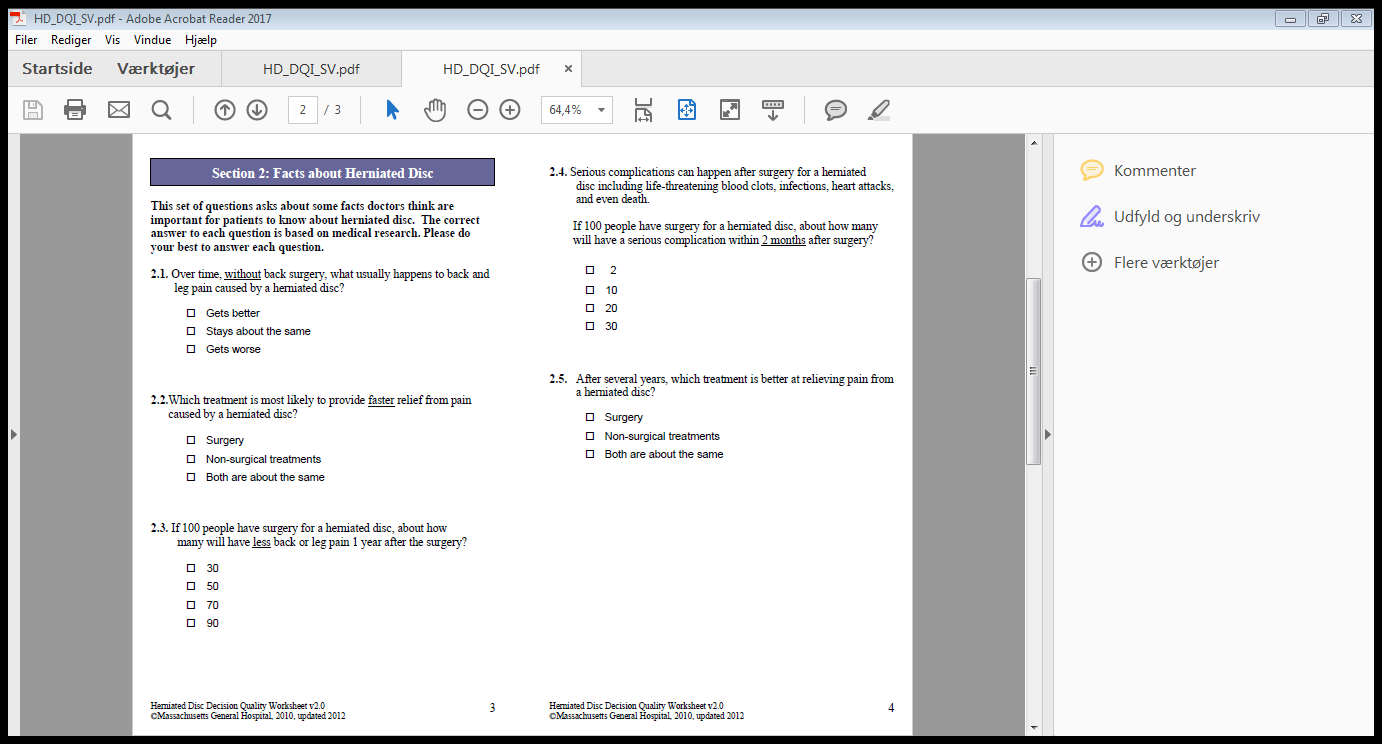


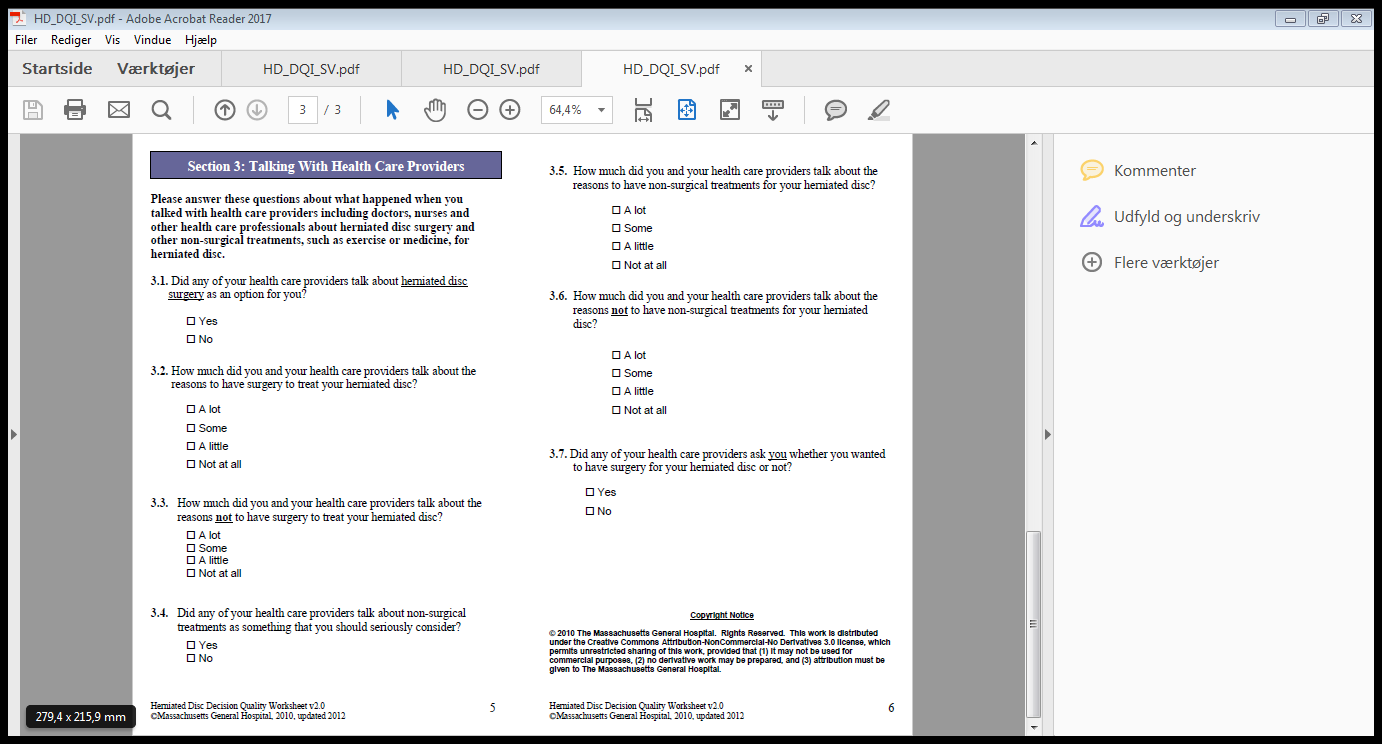


**Decisional Conflict Scale** (DCS) measures personal perception of: uncertainty in choosing options, modifiable factors contributing to uncertainty and effective decision making (feeling the choice is informed, value-based, likely to be implemented)). A total score is calculated ranging from 0-100, [0 = no decisional conflict; 100 = extremely high decisional conflict].

O’connor A. Decisional Conflict Scale. User Manual - Decisional Conflict Scale (16 item statement format). Ottawa: Ottawa Hospital Research Institute, ©1993 [update 2010]. https://decisionaid.ohri.ca/docs/develop/User_Manuals/UM_Decisional_Conflict.pdf. Accessed December 6, 2018.


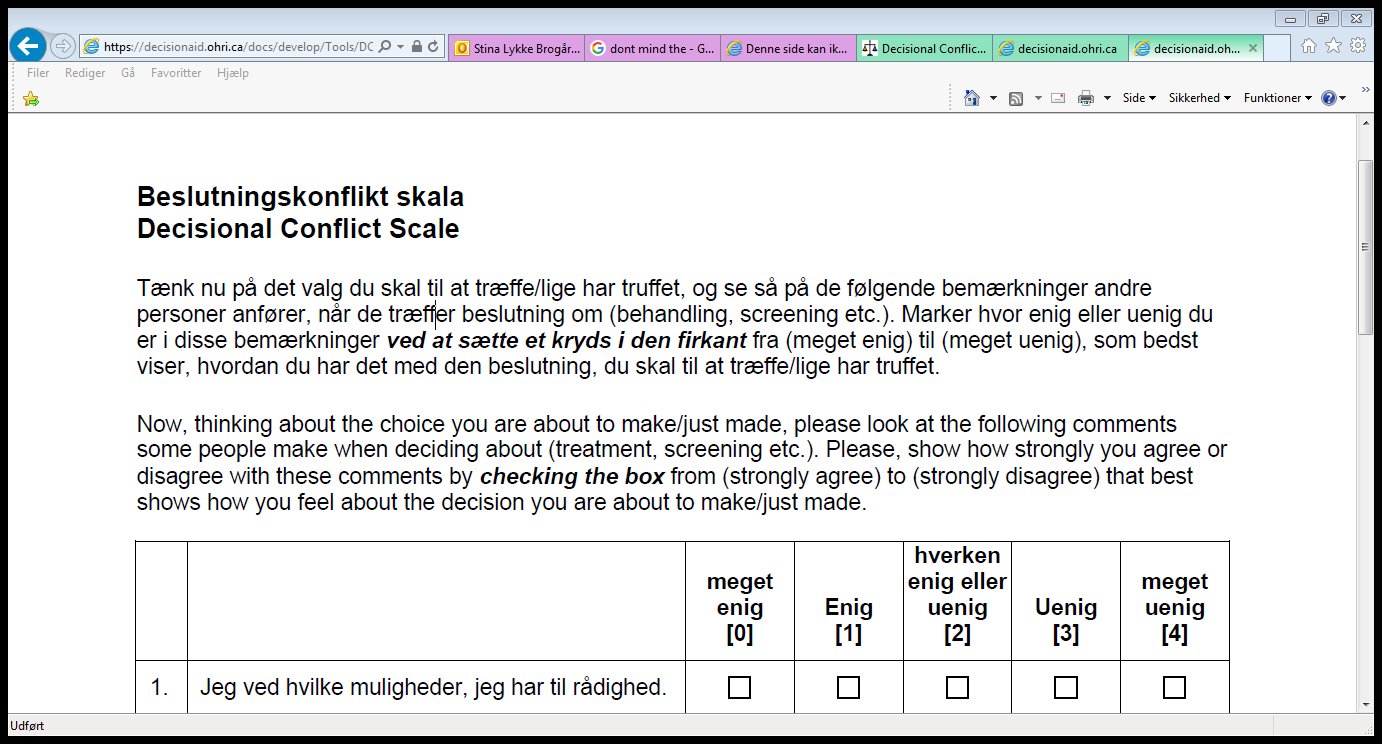


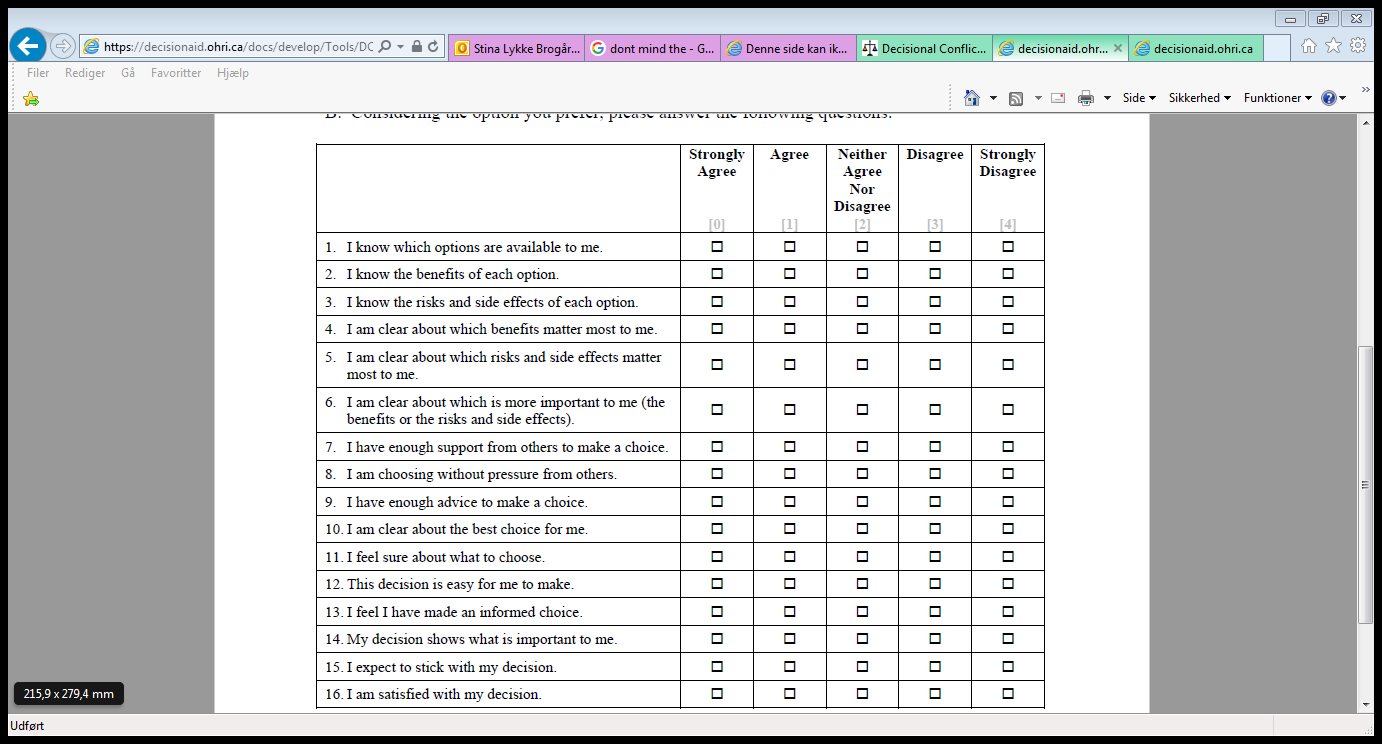


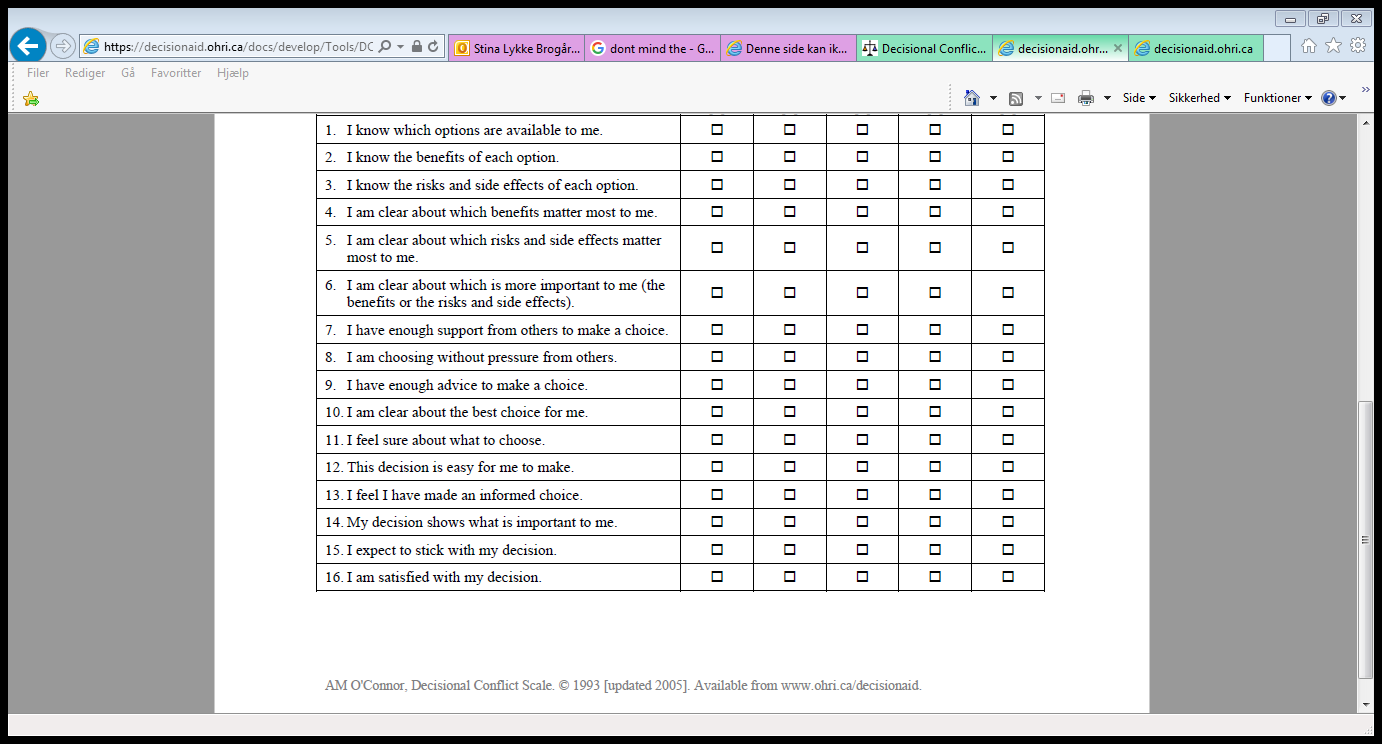


**CollaboRATE** measures patient involvement in the decision making process. A mean score is calculated ranging from 0-9, [1 = No effort was made; 9 = every effort was made].

Elwyn G. CollaboRATE. http://www.glynelwyn.com/uploads/2/4/0/4/24040341/collaborate_for patients_v6.pdf. Accessed December 6, 2018.


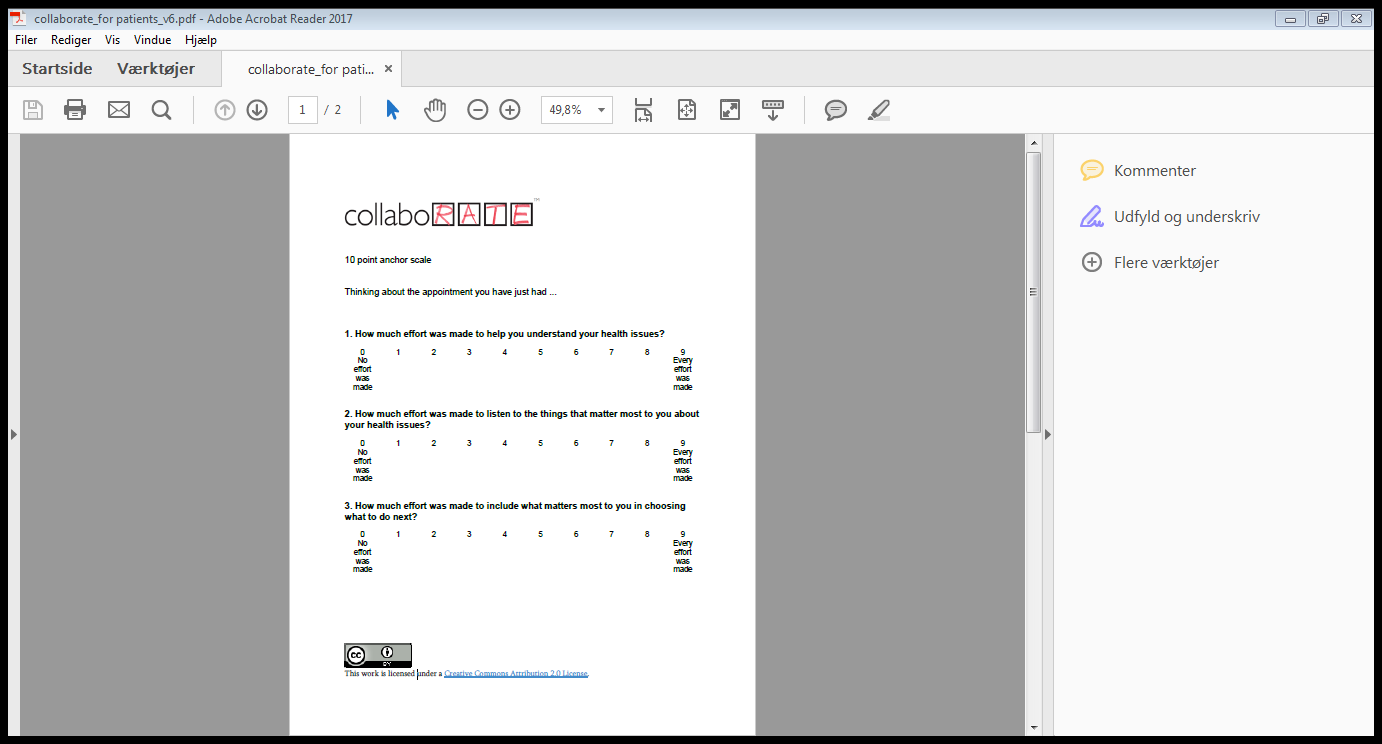

Supplement: Supplementary file 1 — Description and illustration of questionnaires used in this study (DOCX 670 kb) [file 12911_2019_906_MOESM1_ESM.docx]
