# Supplementary material for: Shared decision making when patients consider surgery for lumbar herniated disc: development and test of a patient decision aid
Source: BMC Med Inform Decis Mak. 2019 Oct 4;19:190. doi: 10.1186/s12911-019-0906-9 (PMC6778367; doi:10.1186/s12911-019-0906-9)
Supplement: Supplementary file 2 — Description of Litterature search string and strategy (DOCX 21 kb) [file 12911_2019_906_MOESM2_ESM.docx]

**Appendix II – Search string and strategy for the PtDA content**

We searched Pubmed and The Cochrane Library.

As the surgery technique is constantly changing the search was limited to 10 years. Publication date from 2006/11/01 to 2016/11/01

**Search string in Pubmed** (The string was adapted for Cochrane):

((((((((((lumbago[Title/Abstract]) OR sciatica[Title/Abstract]) OR lumbar herniated disc[Title/Abstract]) OR Intervertebral Disc Degeneration[MeSH Terms]) OR back pain[MeSH Terms]) OR slipped disc[Title/Abstract]) OR "Sciatica"[Mesh]) OR "Pain, Referred"[Mesh]) OR lumbar disc herniation[Title/Abstract]))

AND

((((((((operation[Title/Abstract]) OR spine surgery[Title/Abstract]) OR "Diskectomy, Percutaneous"[Mesh]) OR "Diskectomy"[Mesh]) OR Surgical Procedures, Operative[MeSH Terms]) OR General Surgery[MeSH Terms]))

AND

((((((((("Chiropractic"[Mesh]) OR Manipulation, Chiropractic[MeSH Terms]) OR Physical Therapy Specialty[MeSH Terms]) OR Physical Therapy Modalities[MeSH Terms]) OR Placebos[MeSH Terms]) OR Conservative Treatment[MeSH Terms]) OR Resistance Training[MeSH Terms]) OR Exercise[MeSH Terms]) OR Rehabilitation[MeSH Terms]))

Sort by: Best Match Filters: Publication date from 2006/11/01 to 2016/11/01

*A total of 382 unique records were found in Pubmed 166 trials in Cochrane Library. 73 trials were doublets. Only 1 Cochrane review was found.*

*All titles and if relevant abstract were screened to find out if the study included any of the outcome measures relevant for the PtDA. Full text was read if they met the following criteria:*

- *The study was a randomized controlled trial. Results from reviews of RCT’s were also included*
- *The study compared surgery to non-surgical interventions (i.e. physiotherapy, chiropractic, no treatment etc.).*
- *The patients included were adult (18 years of age) subjects with sciatica due to a herniated disc.*
- *The outcome(s) evaluated included at least one of the outcome measures relevant for the PtDA, (i.e. pain, functional status, recovery or sick leave) using a valid instrument.*
- *The language was limited to English.*

*Exclusion:*

- *If surgical treatment was fusion*
- *If surgical techniques were compared*
